# Functional Alterations of the Basal Ganglia Are Associated with Voluntary Activation of the Core Stabilizing Muscles in Patients with Chronic Low Back Pain: A Cross-Sectional Study

**DOI:** 10.1155/2023/2028379

**Published:** 2023-08-31

**Authors:** Chanjuan Zhang, Xi Chen, Yi Yin, Dongfeng Xie, Jing Luo, Yinan Ai, Wenfeng Zhan, Hongjun Kan, Shuxian Zhang, Guihua Jiang, Xiquan Hu

**Affiliations:** ^1^Department of Rehabilitation Medicine, The Third Affiliated Hospital, Sun Yat-sen University, Guangzhou 510630, China; ^2^Department of Medical Imaging, Guangdong Second Provincial General Hospital, Guangzhou 510317, China

## Abstract

**Purpose:**

Deficits in voluntary activation of the core stabilizing muscles are consistently observed in patients with chronic low back pain (CLBP); however, the underlying neural mechanism remains unclear. This cross-sectional study aimed at testing the hypothesis that the impaired voluntary activation of core stabilizing muscles is associated with structural and functional alterations in the basal ganglia, thalamus, and cortex in patients with CLBP.

**Methods:**

We obtained structural and resting-state functional magnetic resonance imaging (rs-fMRI) data from 53 patients with CLBP and 67 healthy controls and estimated the alterations in grey matter volume (GMV) and functional and effective connectivity (EC) of regions with altered GMV via whole brain analysis. The voluntary activation of the multifidus (MF) and transversus abdominis (TrA) was evaluated by ultrasound imaging in these patients.

**Results:**

Compared with the HCs, they displayed a significant decrease in GMV in the bilateral thalamus and caudate nucleus, a significant increase in GMV in the left middle frontal gyrus, and increased resting-state functional connectivity between the right caudate nucleus and the bilateral precuneus (voxel-level *p* < 0.005, Gaussian random field-corrected *p* < 0.05). The patients also showed increased EC from the right caudate nucleus to the bilateral precuneus, which was significantly correlated with voluntary activation of the bilateral MF and TrA (all *p* < 0.050).

**Conclusions:**

Grey matter alterations may be confined to regions responsible for perception, motor control, and emotion regulation in patients with CLBP. The interrupted EC from the basal ganglia to the default mode network might be involved in the impairment of voluntary activation of the core stabilizing muscles.

## 1. Introduction

Chronic low back pain (CLBP), typically defined as pain below the costal margin and above the inferior gluteal folds, with or without leg pain, has been recognized as the leading cause of disability; it has a mean point prevalence of approximately 12% in the general adult population and imposes a substantial socioeconomic burden [1–4]. Trunk postural control impairment has been suggested to contribute to 90% of CLBP that has no known pathoanatomical causes and no indication for spine surgery [2, 5]. The multifidus (MF) and transversus abdominis (TrA) are among the strongest stabilizing muscles of the lumbar spine and play an essential role in postural control [5]. The voluntary activation of MF and TrA is commonly and reliably estimated by the ultrasound image-measured percent change in muscle thickness [6–8]. Compared with healthy individuals, patients with CLBP show impairments in activation of the MF and TrA that are associated with their impaired postural control [9–12]. Core stabilization exercises use a motor learning approach to improve the function of core stabilizing muscles in patients with CLBP but do not normalize the impaired activation of core stabilizing muscles [5, 13]; thus, the effectiveness of these exercises on CLBP is far from satisfactory [14]. Noninvasive brain stimulation has been suggested to be a promising treatment for CLBP [15], and it improved the effects of exercises in patients with CLBP and other chronic pain syndromes [16]. However, the optimal paradigm of noninvasive brain stimulation in combination with core stabilization exercises for patients with CLBP remains unclear, mainly due to the poor understanding of the neural mechanisms underlying the impaired activation of the MF and TrA. Therefore, unraveling the neural mechanisms of the impaired activation of core stabilizing muscles was essential to initiate noninvasive brain stimulation that could effectively improve activation of core stabilizing muscles in patients with CLBP.

The basal ganglia, thalamus, and primary sensorimotor cortex contribute to human postural control [17], and the basal ganglia plays a central role in the selection of specific muscles to contract and depends on input from the cortex and thalamus [18]. Altered grey matter volume (GMV) and functional connectivity in the basal ganglia, thalamus, primary sensorimotor cortex, etc., were observed in patients with CLBP [19–22]. According to the spinal stability model, postural control is dependent on a constant interplay between the central nervous system and the core stabilizing muscles [5, 23, 24]. The impaired function of core stabilizing muscles could be caused by the neural plasticity of the central nervous system in patients with CLBP [11, 25]. However, it was unknown whether the impaired voluntary contraction of the MF and TrA was associated with structural and functional alterations of the basal ganglia, thalamus, and primary sensorimotor cortex in patients with CLBP.

This study aimed at investigating the relationships between neural alterations and activation of the MF and TrA in patients with CLBP for the first time. We hypothesized that neural alterations of the regions responsible for perception and motor control, such as basal ganglia, thalamus, and primary sensorimotor cortex, were associated with the impaired voluntary contraction of the core stabilizing muscles (the MF and TrA) in patients with CLBP. In order to validate the hypothesis, we performed voxel-based morphometry (VBM) to identify regions with abnormal GMV and analysed resting-state functional connectivity and effective connectivity from the seeds with altered GMV to explore the alterations in the central nervous system as well as their association with the activation of MF and TrA in patients with CLBP.

## 2. Materials and Methods

### 2.1. Study Design

This was a cross-sectional study. The primary outcome was the percent change in thickness of the core stabilizing muscles. The secondary outcomes were the grey matter volume, rsFC, EC, and their correlation with the percent change in thickness of the core stabilizing muscles.

### 2.2. Setting

This study was conducted from September 2019 to October 2022 in the outpatient department of the First Affiliated Hospital of Sun Yat-sen University. Patients with CLBP and HCs were recruited through advertisements.

### 2.3. Participants

The inclusion criteria for patients were as follows: (1) a clinical diagnosis of CLBP with persistent pain >3 months or intermittent pain >6 months [26, 27]; (2) aged 18–65 years [9, 28–30]; (3) score of at least 2 on the Visual Analogue Scale (VAS) in the preceding week [9, 31]; (4) right-hand dominance; (5) absence of neurological diseases (e.g., traumatic brain injury or epilepsy); (6) absence of intracranial lesions; and (7) no pain treatment within the past 3 months.

The exclusion criteria were as follows [9]: (1) radiating pain or low back pain with specific causes (e.g., menstrual pain, vertebral fracture, or severe osteoporosis); (2) presence of cancer, significant unexplained weight loss, cardiocerebrovascular disease, or endocrine disorders; (3) current alcohol/drug dependence or any psychiatric disorders that require current pharmacotherapy; (4) illiteracy/difficulties in communication and/or cognitive deficits (scores <26 Montreal Cognitive Assessment (MoCA)); or (5) any contraindications for MRI (e.g., metal implants in the body).

HCs were selected from participants after applying the exclusion criteria; these participants had no symptoms of low back pain or other pain disorders and were right-handed dominant [9]. The Research Ethics Committee of the First Affiliated Hospital of Sun Yat-sen University (Ethics No. [2019] 408) approved this study. All participants received financial compensation for participating in this study and provided written informed consent after being informed of the purpose and procedures of this study.

### 2.4. Measurement

#### 2.4.1. Clinical Assessments

We used the VAS to assess the average pain intensity in the past week (score range: 0–10; “0” represented no pain, whereas “10” represented unbearable pain), the Short-Form McGill Pain Questionnaire (SFMPQ) to measure each patient's pain experience [32], the Oswestry Disability Index (ODI) [33, 34] to assess low back pain-related disability, and the Pain Catastrophizing Scale (PCS) to assess the extent of catastrophic thinking in response to pain stimuli [35]. Moreover, we used the Hamilton Depression Scale (HAMD) to assess the degree of depression and the MMSE to evaluate cognitive function.

#### 2.4.2. Ultrasound Measurements

**Figure 1:**
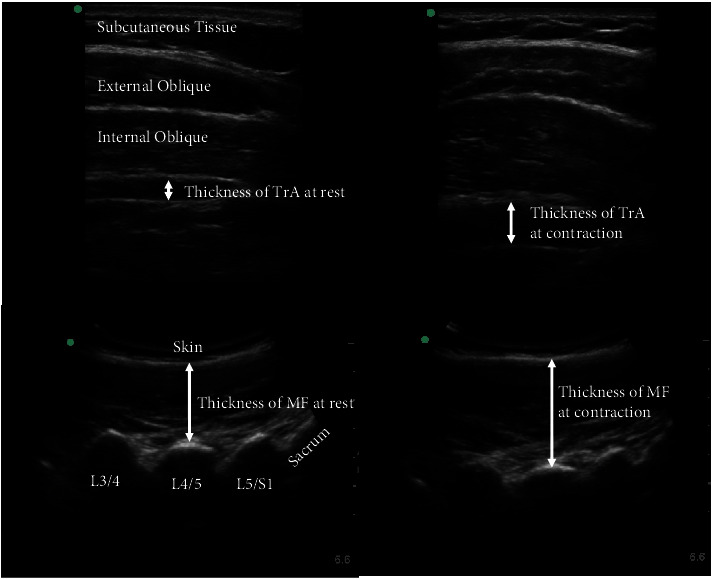
Ultrasound images and measures of MF and TrA. Abbreviations: MF, multifidus; TrA, transversus abdominis.

Ultrasound measurements of all patients with CLBP were taken with Sonosite M-Turbo (B-mode, Seattle, WA, USA) by a single investigator. We positioned a curvilinear transducer (4 MHz) longitudinally at the sacrum level and moved upwards to obtain an image of the MF at the L4-5 zygapophyseal joint (Figure 1). For the measurement of MF at rest, participants lay in the prone position, with a pillow under the abdomen to make the lumbosacral junction angle less than 10°. For the measurement of MF at contraction, participants performed a contralateral arm lift of a small weight to 5 cm above the bed and maintained it at 120° of shoulder abduction and 90° of elbow flexion for approximately 7 seconds until the investigator finished the trial. The weight lifted during the measurement of MF at contraction was determined according to patients' mass: <150 lb (68.2 kg), 1.5 lb (0.7 kg); between 150 and 175 lb (68.2–79.5 kg), 2 lb (0.9 kg); between 175 and 200 lb (79.5–90.9 kg), 2.5 lb (1.1 kg); and 200 lb (90.9 kg), 3 lb (1.4 kg) [7, 36]. We used a linear transducer probe (6–13 MHz) to measure the activation of the TrA and instructed participants to keep a supine crook-lying position (hips flexed to approximately 135° and knees flexed to 90°) at rest and then slowly draw the umbilicus towards the spine and maintained the TrA contraction for 3–5 seconds [7, 9].

All measurements were performed 3 to 5 times bilaterally with a 1-minute rest period and then averaged for analysis. Pictures were exported for offline analysis using ImageJ (version 1.52 k, https://imagej.nih.gov/ij/) by a single examiner. All ultrasound measurements had good reliability [9, 36]. The activation of muscles was calculated as the percent change in thickness by using the following formula:

(1) $$\begin{array}{c} \%Change=\frac{Contraction-Rest}{Rest}\times 100\%\text{.} \end{array}$$

The measuring protocol for the percent change in thickness of TrA and MF showed good test-retest reliability with intraclass correlation coefficient (ICC) values of 0.79–0.99 for both low back pain and healthy subjects [7].

#### 2.4.3. MRI Data Acquisition

We obtained MRI data on a 3.0-T MRI scanner with a 32-channel head coil (Ingenia; Philips, Amsterdam, Netherlands) in the Department of Medical Imaging, Guangdong Second Provincial General Hospital. The participants were instructed to remain motionless with their eyes closed, not to fall asleep, and not to think of anything in particular. The examiner requested that the participants recall their emotions during the rs-MRI scans to evaluate their adherence to the instructions.

High-resolution T1-weighted images were collected with a fast field echo pulse sequence [37] using the following parameters: 185 axial slices, flip angle (FA) = 8°, repetition time (TR) = 7.7 ms, echo time (TE) = 3.5 ms, acquisition matrix = 256 × 256, field of view (FOV) = 256 mm^2^, and slice thickness = 1.0 mm. Upon observing abnormal signs in the T1-weighted images, we obtained T2-fluid-attenuated inversion recovery images to detect brain lesions [37].

Functional MR (fMR) images were collected using a gradient echo-planar imaging (EPI) sequence with the following parameters: 33 transverse slices covering the whole brain, a total of 240 volumes, interleaved scanning, TR = 2,000 ms, TE = 30 ms, acquisition matrix = 64 × 61, FOV = 224 mm × 224 mm, FA = 90°, slice thickness = 3.5 mm, and a 1-mm slice gap.

#### 2.4.4. Structural MRI Data Preprocessing

We performed VBM to detect between-group differences in GMV. We utilized the Computational Anatomy Toolbox (CAT12, version 12.6; https://dbm.neuro.uni-jena.de/cat/) [38], a toolbox implemented within SPM12 (https://www.fil.ion.ucl.ac.uk/spm) running under MATLAB 2013b (MathWorks, Natick, MA, USA), to perform standard preprocessing as follows: data conversion from DICOM to NIFTI format; segmentation into GM, white matter, and cerebrospinal fluid; normalization into standard Montreal Neurological Institute (MNI) space with an isotropic voxel size of 1 mm^3^; and spatial smoothing of the normalized images with an 8-mm full-width at half-maximum (FWHM) Gaussian kernel. An absolute threshold of 0.2 for voxel intensities was applied to minimize partial-volume effects near the border between grey and white matter.

#### 2.4.5. Functional MRI Data Processing

We used DPARSF 3.0 Advanced Edition [39] (https://rfmri.org/DPARSF) based on SPM12 to process the EPI data with the following steps: data format conversion; removal of the first 10 time points; slice timing correction; realignment; coregistration with T1-images, segmentation and DARTEL normalization into standard MNI space with an isotropic voxel size 3 mm × 3 mm × 3 mm; spatial smoothing of the normalized images with a 6-mm FWHM Gaussian kernel; linear detrending and temporal bandpass filtering (0.01–0.1 Hz); and regression analysis to minimize the influence of head motion (Friston 24 model), cerebrospinal fluid, and white matter. We set the head motion reference standard using the mean framewise displacement (FD) Jenkinson and eliminated the participants with motion (mean FD Jenkinson) >2 × standard deviations (SDs) above the group mean motion [40, 41]. Five patients were excluded from the data analysis after realignment preprocessing.

We defined seeds as 6-mm spheres centred on the MNI coordinates of the peak *t* value from regions of abnormal GMV for the seed-based rsFC and Granger causality analysis (GCA).

#### 2.4.6. Seed-Based rsFC Analysis

We calculated Pearson's correlation coefficients of time-series data extracted from all voxels in those seeds and the other voxels in the whole brain. Then, the rsFC maps were converted to z-rsFC maps to improve the normality of the data distribution by Fisher's z-transformation.

#### 2.4.7. Seed-Based GCA

We applied Granger causal connectivity to determine the EC of the time series of the seed regions with abnormal GMV to other voxels in the whole brain (*X* to *Y*) and the EC of the time series of other voxels in the whole brain to those predefined seeds (*Y* to *X*). In Granger's principle, connectivity from *X* to *Y* signifies that *X* has a “causal influence” on *Y*; in other words, neuronal activity in *X* precedes and predicts neuronal activity in *Y* and vice visa [42].

Bivariate coefficient GCA was conducted to estimate the strength and direction of the relationship between the predefined seeds (*X*) and the rest of the brain (*Y*) by seed-to-whole-brain analysis performed in RESTplus software (RESTplus v1.24; https://restfmri.net/forum/restplus). The GCA maps were converted to z-GCA maps by Fisher's z-transformation.

#### 2.4.8. Study Size

Sample size was calculated using the G∗Power statistical software (version 3.1.2; https://gpower.hhu.de) based on the effect size of 0.599 calculated from our previous study for percent change in thickness of TrA between patients with CLBP (88.754 ± 33.823) and HCs (45.628 ± 22.722) [9] (test family: *t*-tests, statistical test: means, difference between two independent groups (two groups), type of power analysis: a priori, allocation ratio = 1 : 1) [43]. A minimum of 52 patients in each group was required, assuming an *α* level of 0.05 and a power (1-beta) of 0.85. Considering a dropout rate of 20% after quality control of MRI data, the minimum number of patients for enrolment was set to 65.

#### 2.4.9. Statistical Methods

We used SPM 12 implemented in MATLAB2013b to perform two independent-sample *t* tests to estimate the between-group differences in (1) GMV with age, sex, and total brain volume as covariates and (2) rs-fMRI data (rsFC and GCA) with age, sex, and head motion (mean FD following Jenkinson) as covariates. Here, we applied a data-driven approach to identify the clusters of significant between-group differences by performing all the analyses within the whole-brain mask. The multiple comparisons of all the MRI data were corrected by the voxel-level *p* < 0.005 [44, 45] followed by cluster-level Gaussian random field (GRF)-corrected *p* < 0.05. The locations of statistically significant clusters and the corresponding MNI coordinates were identified by xjView 8.8 (https://www.alivelearn.net/xjview8) based on SPM12 running under MATLAB 2013b. DPARSF 3.0 was utilized to extract the mean VBM, rsFC, and GCA values of the significant regions (averaged across all voxels in each cluster).

Statistical analysis was performed using SPSS, version 26.0 (SPSS Inc. Chicago, IL, USA). The continuous variables in each group were assessed for normality and homogeneity by the Kolmogorov‒Smirnov test and Levene's test, respectively. The age, education length, HAMD and MoCA scores in both groups, scores of VAS, SFMPQ, and PCS, subscores of SFMPQ and PCS, and the BMI in the CLBP group were not normally distributed. The BMI in the HC group, the ODI scores, and the percent change in thickness of the core stabilizing muscles in the CLBP group, and the extracted values of grey matter volume of significant between-group differences, the GCA, and the FC were normally distributed. Mann‒Whitney *U* tests were conducted to determine the differences in the age, education length, BMI, and scores of ODI, HAMD, and MoCA between the CLBP and HC groups according to the distribution of variables. Using partial correlation analyses according to the distribution of variables, we examined the associations between clinical parameters and (1) abnormal structural metrics (with age, sex, and total brain volume as covariates) and between clinical parameters and (2) functional metrics (with age, sex, and head motion as covariates), all of which were corrected for multiple comparisons using the Bonferroni correction. The associations between ODI scores and the percent change in thickness of the core stabilizing muscles and the abnormal structural and functional metrics were examined by Pearson partial correlation analyses, while the associations between the scores of VAS, SFMPQ, and PCS, the subscores of SFMPQ and PCS, and the abnormal structural and functional metrics were examined by Spearman partial correlation analyses. The significance threshold was set at *p* < 0.05.

## 3. Results

### 3.1. Demographic and Clinical Characteristics

**Figure 2:**
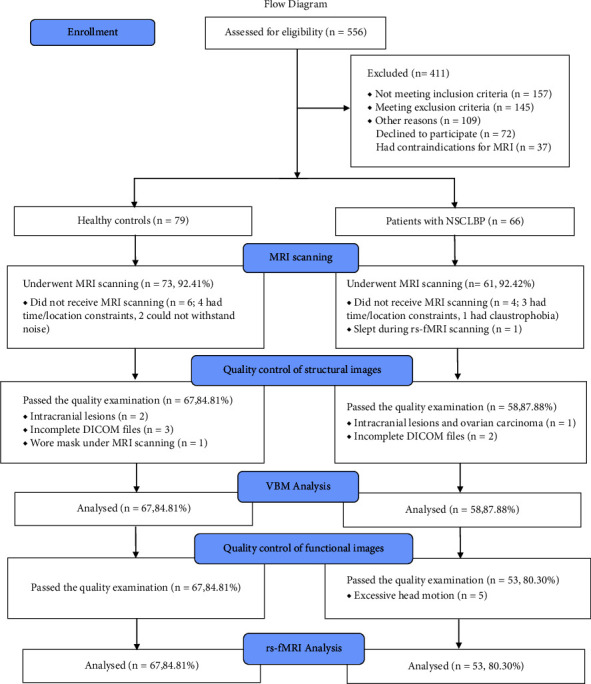
The flow diagram. Abbreviations: CLBP, chronic low back pain; rs-fMRI, resting-state functional magnetic resonance imaging.

We recruited 145 eligible participants. Twenty participants were excluded for the following reasons: (i) seven did not participate because of time and location constraints; (ii) two refused to undergo MRI scans because of intolerance of the scanning noise; (iii) one had claustrophobia; (iv) one fell asleep during the rs-fMRI scan; (v) three had intracranial lesions with evidence of abnormal signs in T1-weighted sequences that were confirmed to be T2-hyperintense lesions, including one diagnosed with ovarian carcinoma; (vi) five had incomplete DICOM files; (vii) one underwent MRI scans with a mask on the face; and (viii) five patients were excluded from the analysis of rs-fMRI data due to excessive head motion. Eventually, we included 53 patients and 67 HCs in the analysis (Figure 2).

The two groups did not significantly differ in terms of age, sex, body mass index, weight, height, or years of education (all *p* > 0.05). Notably, the CLBP patients scored distinctly higher on the HAMD than the HCs (*p* < 0.001).  Table 1 presents the participants' demographic and clinical characteristics.

### 3.2. Decreased Grey Matter Volume in CLBP Patients

**Figure 3:**
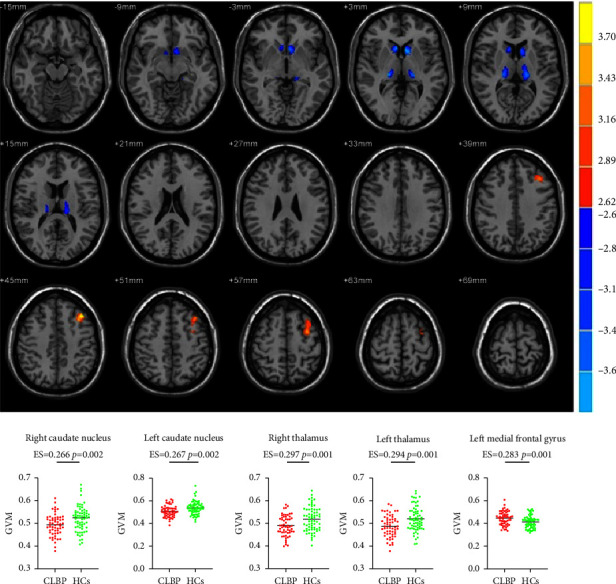
Between-group differences in GMV. Patients with CLBP displayed a significant decrease in GMV in the bilateral thalamus and caudate nucleus and a significant increase in GMV in the left middle frontal gyrus. Abbreviations: ES, effect size; GMV, grey matter volume; HCs, healthy controls; and CLBP, chronic low back pain.

The CLBP group exhibited decreased GMV in the bilateral caudate nucleus and thalamus and increased GMV in the left middle frontal gyrus (uncorrected voxel-level *p* < 0.005, cluster-level GRF-corrected *p* < 0.05) (Figure 3 and Table 2).

### 3.3. Increased Resting-State Functional Connectivity in Patients

**Figure 4:**
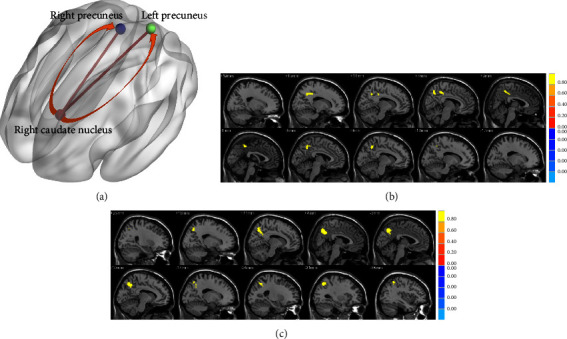
Between-group differences in rsFC and GCA. (a) With the right caudate nucleus as a seed, the rsFC between the right caudate nucleus and the bilateral precuneus, and the EC from the right caudate nucleus to the bilateral precuneus were significantly increased in patients with CLBP. (b) The between-group difference in rsFC between the right caudate nucleus and the whole brain. (c) The between-group difference in EC from the right caudate nucleus to the whole brain. Abbreviations: CLBP, chronic low back pain; EC, effective connectivity; GCA, Granger causality analysis; rsFC, resting-state functional connectivity.

The CLBP patients exhibited increased rsFC between the right caudate nucleus (one of the seeds with abnormal GMV) and the bilateral precuneus (voxel-level *p* < 0.005, GRF-corrected *p* < 0.05) compared with HCs (Figure 4 and Table 3).

### 3.4. Increased EC from the Right Caudate Nucleus to the Bilateral Precuneus in Patients

We observed significantly increased EC from the right caudate nucleus (one of the seeds with abnormal GMV) to the bilateral precuneus (voxel-level *p* < 0.005, cluster-level GRF-corrected *p* < 0.05) (Figure 4 and Table 4).

### 3.5. Correlations between the fMRI Data and the Clinical Characteristics

**Figure 5:**
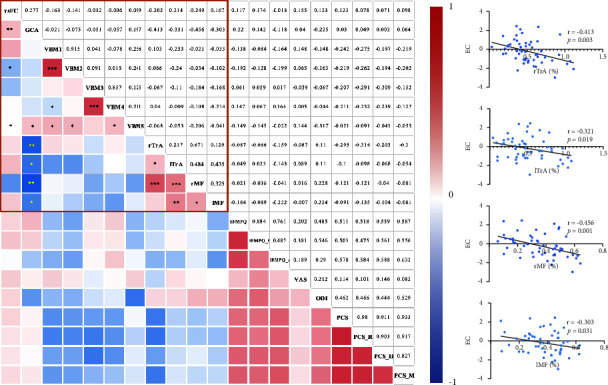
Correlations of the brain structural and functional alterations with the voluntary contraction of the core stabilizing muscles and clinical assessments. The EC from the right caudate nucleus to the bilateral precuneus was significantly correlated with the voluntary contraction of the bilateral core stabilizing muscles in patients with CLBP. Abbreviations: CLBP, chronic low back pain; EC, effective connectivity from the right caudate nucleus into the bilateral precuneus; GMV1, grey matter volume of the right caudate nucleus; GMV2, grey matter volume of the left caudate nucleus; GMV3, grey matter volume of the right thalamus; GMV4, grey matter volume of the left thalamus; GMV5, grey matter volume of the left middle frontal gyrus; lMF, left multifidus; lTrA, left transversus abdominus; rMF, right multifidus; rTrA, right transversus abdominus; ODI, Oswestry disability index; PCS, pain catastrophizing scale; PCS_H, helplessness subscale of the pain catastrophizing scale; PCS_M, magnification subscale of the pain catastrophizing scale; PCS_R, rumination subscale of the pain catastrophizing scale; rsFC, resting-state functional connectivity; SFMPQ, short form of the McGill pain questionnaire; SFMPQ_A, affective subscale of the short form of the McGill pain questionnaire; SFMPQ_S, sensory subscale of the short form of the McGill pain questionnaire; and VAS, visual analogue scale. ^*∗*^*p* < 0.05, ^*∗∗*^*p* < 0.01, and ^*∗∗∗*^*p* < 0.001.

We found significantly negative correlations of EC from the right caudate nucleus to the bilateral precuneus with voluntary activation of the left TrA (*r* = −0.322, *p*=0.021), the right TrA (*r* = −0.412, *p*=0.003), the left MF (*r* = −0.303, *p*=0.031), and the right MF (*r* = −0.456, *p*=0.001) (Figure 5).

Partial correlation analyses showed a correlation between the mean GMV of the right thalamus and PCS rumination (*r* = −0.266, *p*=0.049) and between the mean GMV of the right thalamus and helplessness scores (*r* = −0.291, *p*=0.031). However, none of those correlations remained significant after Bonferroni correction (*p* < 0.001).

## 4. Discussion

To the best of our knowledge, this study is among the first to estimate the associations of the impaired voluntary contraction of core stabilizing muscles with structural and functional plasticity of the brain in patients with CLBP. We observed decreased GMV in the bilateral caudate nucleus and thalamus, increased GMV in the left middle frontal gyrus, increased rsFC between the right caudate nucleus and the bilateral precuneus, and increased EC from the right caudate nucleus to the bilateral precuneus. The altered EC was significantly correlated with the voluntary activation of the core stabilizing muscles.

### 4.1. Structural Abnormalities in the CLBP Group

In this study, we found decreased GMV in the bilateral caudate nucleus and thalamus in the CLBP patients, which was similar to the findings of studies that included patients with specific and nonspecific chronic low back pain [20, 46] and other chronic syndromes [47–49]. Another study found increased grey matter in the thalamus in patients with CLBP [50]. The discrepancy may be due to the use of different thresholds for multiple comparison correction, as the previous study utilized a lower threshold (uncorrected *p* < 0.001) [50] that could lead to false positives [51, 52]. Interestingly, there were no significant alterations in GMV [53] or increased GMV in the bilateral putamen and nucleus accumbens or right caudate nucleus in patients with CLBP [54] reported by previous studies; participants in these studies might have had specific or nonspecific CLBP. In the present study, we only included patients with nonspecific CLBP, and the effect size (>0.26) of the altered GMV was much larger than that in the previous study (effect size = 0.07) in normalized whole-brain volume between the groups, which would contribute to the discrepancies. Notably, the small Gaussian kernel (3-mm FWHM) used for spatial smoothing of the normalized images in the previous study [54] could also account for the discrepancies.

The thalamus is critical for translating nociceptive inputs to the cortex and plays an instrumental role in motor activity, emotion, and other sensorimotor association functions [55]. The caudate nucleus is an important structure in the basal ganglia that receives inputs from all cortical areas and, through the thalamus, projects primarily to frontal lobe areas [55]. The motor basal ganglia loop is central to motor control, and the nonmotor basal ganglia loops are involved in pain, sensory integration, visual processing, cognition, and emotion [56]. The basal ganglia-thalamic-cortical loop integrates many aspects of pain, including the integration of motor, emotional, autonomic, and cognitive responses to pain [57]. The decreased GMV of the bilateral caudate nucleus and thalamus may thus explain the impaired postural control and negative cognitive-emotional responses to pain, such as pain catastrophizing [9], and reflect the consequence of constant input of afferent nociceptive information in CLBP patients [20, 50].

CLBP patients also exhibited increased GMV in the left middle frontal gyrus, which is one of the key regions for emotion regulation. Increased thickness of the middle frontal gyrus (specifically, the rostral middle frontal gyrus) is positively associated with perceived stress and sadness [58]. Similar to the common clinical observation that chronic pain syndromes are comorbid with psychological disorders, patients with CLBP usually also suffer from psychological distress, such as depression, pain catastrophizing [9], and stress [59]. We assumed that the increased GMV in the left middle frontal gyrus might be a consequence of the stress that accompanies CLBP. However, we did not evaluate stress in this study. The association between the increased GMV in the middle frontal gyrus and stress among these patients needs to be verified in the future.

### 4.2. Functional Brain Alterations in Patients with CLBP

Patients with CLBP show increased activity in superficial back muscles and reduced activation in core stabilizing muscles [9, 60, 61]. These changes might reflect alterations in the postural control strategy adopted by the nervous system [61]. In this study, patients with CLBP showed increased rsFC between the right caudate nucleus and the bilateral precuneus and increased EC from the right caudate nucleus to the bilateral precuneus, which were negatively associated with the voluntary activation of the bilateral core stabilizing muscles. The caudate nucleus, a fundamental structure of the basal ganglia largely responsible for motor function, such as voluntary movement and action selection, is crucial for planning and performing tasks necessary to achieve complex goals [18, 47, 62]. The precuneus is the core hub of the default mode network, which is crucial for attention, memory, introspection, and self-referential processes [63] and is possibly involved in the assessment and integration of pain [64]. The enhanced rsFC between the basal ganglia (the caudate nucleus) and the default mode network (the precuneus) may reflect the “tight” motor control phenotype of the superficial back muscles in patients with CLBP by causing muscular hyperactivity as a “guarding strategy” due to the overestimation of the threat or severity of painful stimuli [65, 66] and because of the reduced activation in core stabilizing muscles [9, 60, 61]. However, we did not estimate the relationship between abnormal rsFC and activation of the superficial back muscles; this association requires further investigation. The onset of core stabilizing muscles' activation during postural control tasks was associated with the reorganization of core stabilizing muscles representation at the motor cortex in patients with CLBP [11, 25]. We found a negative association of the EC from the basal ganglia (the caudate nucleus) to the default mode network (the precuneus) with the voluntary activation of the core stabilizing muscles, providing further evidence for the neural mechanisms of impaired trunk postural control in patients with CLBP. Thus, noninvasive brain stimulations that could directly influence the function of the basal ganglia might improve the voluntary activation of core stabilizing muscles in patients with CLBP more effectively than controversial exercise treatments, which needs to be verified in future studies.

Mao et al. [21] observed increased rsFC of the right thalamomotor/somatosensory pathway in patients with CLBP by setting the motor/somatosensory cortex as seeds; this change was significantly and positively correlated with the ongoing pain intensity during the rs-fMRI scan. As brain functional images are sensitive to participants' ongoing state, we deduced that the pain intensity during the rs-fMRI scan could influence the results of rs-fMRI analysis. However, we did not assess pain intensity during the rs-fMRI scan, which prevented us from performing further analysis. Future studies are needed to determine the effects of ongoing pain intensity on the functional characteristics of patients with CLBP.

## 5. Limitations

Nevertheless, the findings of the present study should be interpreted with caution due to some limitations. First, the relationship between the structural and functional metrics and the clinical assessments was not straightforward. Second, we observed abnormal GMV, rsFC, and EC from the basal ganglia but did not estimate the relationship between those abnormalities and postural control, which requires further clarification. Additional studies that include measurements of postural control may allow us to gain a deeper understanding of the neural mechanisms of impaired postural control in people with CLBP.

## 6. Conclusions

Our study demonstrated that patients with CLBP had grey matter atrophy in structures specific to perception and motor control, such as the thalamus and caudate nucleus. Furthermore, the disrupted EC from the basal ganglia to the default mode network might be involved in the impaired voluntary activation of the core stabilizing muscles. These results provided preliminary evidence that the functional alteration of the basal ganglia might contribute to the impaired core stabilizers of the lumbar spine in patients with CLBP.

## Figures and Tables

**Table 1:** Characteristics of participants in the analysis of rs-fMRI data.

Characteristics	HC (*n* = 67)	CLBP (*n* = 53)	*p* value
Male/Female (*n*)^†^	23/44	20/33	0.669
Median age, *y* (range)	27 (21–60)	26 (20–50)	0.683
Median BMI (kg/m^2^) (range)	21.107 (17.58–25.71)	20.861 (17.502–29.862)	0.617
Median education length, *y* (range)	18 (8–22)	18 (11–23)	0.346
Median pain duration, *m* (range)	N/A	24 (4–192)	N/A
Median HAMD (range)	0 (0–5)	3 (0–6)	<0.001
Median MoCA (range)	29 (27–30)	29 (27–30)	0.066
Median VAS (0–10 cm)	N/A	6 (2.4–8)	N/A
Median ODI (%) (range)	N/A	14 (0–33.33)	N/A
Median PCS (range)	N/A	14 (1–33)	N/A
Median PCS_R (range)	N/A	6 (0–21)	N/A
Median PCS_M (range)	N/A	4 (0–15)	N/A
Median PCS_H (range)	N/A	3 (0–15)	N/A
Median SFMPQ (range)	N/A	8 (2–24)	N/A
Median SFMPQ_A (range)	N/A	3 (0–8)	N/A
Median SFMPQ_S (range)	N/A	4 (1–16)	N/A
Median rTrA% (range)	N/A	48.400 (3.300–111.465)	N/A
Median lTrA% (range)	N/A	47.500 (4.30–107.90)	N/A

Abbreviations: BMI, body mass index; CLBP, chronic low back pain; HAMD, Hamilton depression scale; HCs, healthy controls; Montreal cognitive assessment (MoCA); ODI, Oswestry disability index; PCS, pain catastrophizing scale; PCS_H, helplessness subscale of the pain catastrophizing scale; PCS_M, magnification subscale of the pain catastrophizing scale; PCS_R, rumination subscale of the pain catastrophizing scale; SFMPQ, short-form McGill pain questionnaire; SFMPQ_A, affective subscale of the short-form McGill pain questionnaire; SFMPQ_S, sensory subscale of the short-form McGill pain questionnaire; VAS, visual analogue scale; N/A, not applicable. ^†^Chi-square.

**Table 2:** Brain areas with altered GMV in the CLBP group.

Brain area	MNI coordinates			*T* value	Cluster size (clusters)
	*x*	*y*	*z*		
Left caudate nucleus	−6	13.5	1.5	−3.7795	753
Right caudate nucleus	7.5	16.5	4.5	−3.7697	387
Left thalamus	−18	−27	6	−3.7489	680
Right thalamus	21	−25.5	3	−3.944	458
Left medial frontal gyrus	−39	25.5	46.5	3.9754	1019

Abbreviations: MNI, Montreal neurologic institute; CLBP, chronic low back pain; GMV, grey matter volume.

**Table 3:** Between-group differences in seed-based rsFC analysis (patients > HCs).

Seed	Brain area	MNI coordinates			*T* value	Cluster size (clusters)
		*x*	*y*	*z*		
Right caudate nucleus	Bilateral precuneus	−6	−66	45	3.8161	383

Abbreviations: CLBP, chronic low back pain; HCs, healthy controls; MNI, Montreal neurologic institute; rsFC, resting-state functional connectivity.

**Table 4:** Between-group differences in seed-based GCA (patients > HCs).

Effective connectivity from the right caudate nucleus to the rest of the brain					
Brain area	Peak MNI coordinates (*x*, *y*, *z*)	Mean (SD) path coefficient		*T* values (peak)	Cluster size (voxels)
		CLBP	HCs		
Bilateral precuneus	15, −54, 39	−0.219 (1.210)	−1.008 (1.018)	3.7961	120

Abbreviations: CLBP, chronic low back pain; GCA, Granger causality analysis; HCs, healthy controls; and MNI, Montreal neurological institute.

## Data Availability

The raw data used to support the findings of this study are included within the supplementary information file.

## Abbreviations

CLBP: Chronic low back pain
EC: Effective connectivity
EPI: Echo-planar imaging
ES: Effective size
FA: Flip angle
FD: Framewise displacement
FDR: False discovery rate
FFE: Fast field echo
fMR: Functional magnetic resonance
FOV: Field-of-view
FWHM: Full-width at half-maximum
GCA: Granger causality analysis
GMV: Grey matter volume
GRF: Gaussian random field
HAMD: Hamilton depression scale
HCs: Healthy controls
lMF: Left multifidus
lTrA: Left transversus abdominis
rMF: Right multifidus
rTrA: Right transversus abdominis
rs-fMRI: Resting-state functional magnetic resonance imaging
rsFCs: Resting-state functional connectivity
MF: Multifidus
MoCA: Montreal cognitive assessment
MNI: Montreal neurological institute
ODI: Oswestry disability index
PCS: Pain catastrophizing scale
PCS_H: Helplessness subscale of the pain catastrophizing scale
PCS_M: Magnification subscale of the pain catastrophizing scale
PCS_R: Rumination subscale of the pain catastrophizing scale
SDs: Standard deviations
SFMPQ: Short-form McGill pain questionnaire
SFMPQ_A: Affective subscale of the short form of the McGill pain questionnaire
SFMPQ_S: Sensory subscale of the short form of the McGill pain questionnaire
TE: Echo time
TR: Repetition time
TrA: Transversus abdominis
VAS: Visual analogue scale
VBM: Voxel-based morphometry.
