# Supplementary material for: Functional Alterations of the Basal Ganglia Are Associated with Voluntary Activation of the Core Stabilizing Muscles in Patients with Chronic Low Back Pain: A Cross-Sectional Study
Source: Pain Res Manag. 2023 Aug 31;2023:2028379. doi: 10.1155/2023/2028379 (PMC10484657; doi:10.1155/2023/2028379)
Supplement: Supplementary Materials — Data of this paper can be found in the supplementary material. [file 2028379.f1.doc]

Group	mean_FD_	Pain_Dur	MoCA	HAMD	Age	Gender	Height	Weight	BMI	
1	0.059848	10	29	6	26	1	1.66	62.5	22.68109	
1	0.061664	25	29	6	50	1	1.57	52	21.09619	
1	0.050644	60	28	6	27	1	1.62	58	22.10029	
1	0.069169	84	29	3	25	1	1.69	60	21.00767	
1	0.101059	12	29	6	23	1	1.62	50	19.05197	
1	0.052025	24	30	4	26	1	1.56	61	25.06575	
1	0.062507	72	27	3	35	0	1.8	72	22.22222	
1	0.064895	96	29	6	30	0	1.7	59	20.41522	
1	0.082598	24	29	1	31	0	1.72	74	25.01352	
1	0.136273	10	27	6	28	1	1.55	43	17.89802	
1	0.104924	60	29	5	46	1	1.66	74	26.85441	
1	0.057753	12	29	5	22	1	1.55	44	18.31426	
1	0.07297	36	29	0	23	0	1.83	66	19.70796	
1	0.063056	120	29	3	24	1	1.62	50	19.05197	
1	0.060127	12	29	2	28	0	1.64	60	22.30815	
1	0.077052	24	29	4	20	0	1.78	76	23.98687	
1	0.099893	10	28	0	27	0	1.75	62	20.2449	
1	0.088842	24	28	2	25	1	1.59	50	19.7777	
1	0.089141	24	27	4	23	1	1.6	50	19.53125	
1	0.080095	6	29	3	36	1	1.55	65	27.05515	
1	0.080103	144	27	6	47	1	1.58	51	20.42942	
1	0.045606	6	29	6	25	1	1.575	47	18.94684	
1	0.051361	18	29	0	25	1	1.6	47	18.35938	
1	0.13155	60	29	0	21	0	1.69	61	21.3578	
1	0.077235	36	29	5	22	1	1.63	47	17.68979	
1	0.069698	12	28	0	25	1	1.61	47	18.13202	
1	0.090698	120	29	0	31	0	1.78	71	22.40879	
1	0.092732	14	27	0	24	1	1.56	50	20.54569	
1	0.083343	48	28	6	23	0	1.8	75	23.14815	
1	0.105214	36	28	1	34	1	1.56	45	18.49112	
1	0.088205	48	29	1	29	1	1.72	65	21.97134	
1	0.070597	120	28	0	50	0	1.68	65	23.03005	
1	0.054134	72	29	2	25	0	1.73	60	20.04745	
1	0.103864	96	28	0	37	0	1.76	92.5	29.86183	
1	0.075772	192	27	1	38	1	1.65	56	20.56933	
1	0.09086	24	28	3	23	0	1.79	61	19.03811	
1	0.055143	10	27	6	24	1	1.65	57	20.93664	
1	0.125875	24	29	0	25	0	1.78	73	23.04002	
1	0.090374	4	29	0	24	0	1.64	70	26.02617	
1	0.081383	12	28	5	29	1	1.6	50	19.53125	
1	0.077559	50	29	4	36	0	1.65	64	23.50781	
1	0.056937	6	30	0	22	1	1.69	72	25.2092	
1	0.159157	24	29	2	23	1	1.63	46.5	17.5016	
1	0.071791	6	29	1	33	0	1.8	72	22.22222	
1	0.065882	60	29	4	23	1	1.65	50	18.36547	
1	0.088842	108	29	5	32	0	1.64	68	25.28257	
1	0.057916	6	29	3	27	1	1.6	49	19.14063	
1	0.069653	36	29	3	34	0	1.71	61	20.86112	
1	0.096613	36	29	2	25	1	1.55	55	22.89282	
1	0.046193	12	29	2	28	1	1.65	52	19.10009	
1	0.071609	12	29	1	30	1	1.63	56	21.0772	
1	0.05194	26	29	5	27	1	1.6	53	20.70313	
1	0.084693	12	28	1	23	1	1.65	53	19.4674	


0	0.041227	#NULL!	28	0	29	1	1.53	42	17.94182	
0	0.146038	#NULL!	29	0	43	1	1.635	47	17.58176	
0	0.041227	#NULL!	30	0	25	0	1.8	60	18.51852	
0	0.042203	#NULL!	28	0	23	1	1.64	62	23.05175	
0	0.052972	#NULL!	28	0	43	1	1.63	57	21.45357	
0	0.055906	#NULL!	28	0	37	0	1.76	70	22.59814	
0	0.054955	#NULL!	28	0	24	1	1.56	45	18.49112	
0	0.073197	#NULL!	29	0	26	0	1.81	71.9	21.94683	
0	0.071411	#NULL!	29	0	23	0	1.76	69	22.27531	
0	0.048297	#NULL!	29	5	26	1	1.6	52	20.3125	
0	0.075025	#NULL!	28	0	44	1	1.6	60	23.4375	
0	0.08568	#NULL!	30	0	44	1	1.55	55	22.89282	
0	0.086951	#NULL!	30	3	22	1	1.5	55	24.44444	
0	0.063549	#NULL!	29	3	23	1	1.58	53	21.23057	
0	0.08694	#NULL!	30	0	24	0	1.7	65	22.49135	
0	0.055435	#NULL!	30	1	22	1	1.62	55	20.95717	
0	0.074597	#NULL!	28	0	29	1	1.6	63	24.60938	
0	0.06182	#NULL!	29	0	30	0	1.65	60	22.03857	
0	0.070729	#NULL!	29	0	21	0	1.71	54	18.46722	
0	0.064189	#NULL!	30	0	25	1	1.6	54	21.09375	
0	0.108627	#NULL!	30	0	28	0	1.62	56	21.33821	
0	0.056387	#NULL!	28	0	60	1	1.62	53	20.19509	
0	0.056893	#NULL!	28	0	26	1	1.7	70	24.22145	
0	0.056218	#NULL!	28	0	25	1	1.61	53	20.44674	
0	0.069615	#NULL!	29	0	32	0	1.76	78	25.18079	
0	0.063838	#NULL!	29	0	27	0	1.73	65	21.71807	
0	0.070987	#NULL!	29	0	34	0	1.78	60	18.937	
0	0.058039	#NULL!	29	0	28	0	1.81	83	25.335	
0	0.062664	#NULL!	29	0	24	1	1.6	50	19.53125	
0	0.055439	#NULL!	29	0	26	1	1.65	52	19.10009	
0	0.053594	#NULL!	30	0	27	0	1.75	65	21.22449	
0	0.050499	#NULL!	27	0	28	1	1.68	51	18.06973	
0	0.068701	#NULL!	30	0	23	1	1.64	52	19.33373	
0	0.057472	#NULL!	29	0	26	0	1.77	64	20.42836	
0	0.045656	#NULL!	29	0	25	1	1.63	50	18.81892	
0	0.05909	#NULL!	28	0	25	0	1.71	70	23.93899	
0	0.036336	#NULL!	30	0	24	1	1.68	55	19.48696	
0	0.060322	#NULL!	28	0	31	0	1.55	52	21.64412	
0	0.056277	#NULL!	28	0	24	1	1.5	40	17.77778	
0	0.064511	#NULL!	28	0	29	0	1.68	70	24.80159	
0	0.06783	#NULL!	29	0	45	1	1.6	65	25.39063	
0	0.041526	#NULL!	29	0	29	1	1.6	56	21.875	
0	0.065004	#NULL!	28	2	31	0	1.77	73	23.30109	
0	0.058457	#NULL!	29	0	24	1	1.65	70	25.71166	
0	0.055442	#NULL!	29	0	36	1	1.63	50	18.81892	
0	0.041569	#NULL!	28	0	32	1	1.62	55	20.95717	
0	0.077101	#NULL!	28	0	29	1	1.62	58	22.10029	
0	0.039557	#NULL!	28	0	24	1	1.68	60	21.2585	
0	0.035834	#NULL!	30	0	27	0	1.69	53	18.55677	
0	0.041335	#NULL!	29	0	29	1	1.7	53	18.3391	
0	0.077764	#NULL!	29	0	28	1	1.53	60	25.63117	
0	0.065829	#NULL!	28	0	25	1	1.66	56	20.32225	
0	0.059405	#NULL!	29	1	29	1	1.58	47	18.82711	
0	0.074657	#NULL!	29	0	29	1	1.6	65	25.39063	


0	0.070411	#NULL!	28	0	27	1	1.58	63	25.23634	
0	0.057903	#NULL!	28	0	24	1	1.5	47	20.88889	
0	0.052527	#NULL!	29	0	28	0	1.67	58	20.79673	
0	0.064786	#NULL!	28	0	30	1	1.65	52	19.10009	
0	0.074846	#NULL!	30	0	24	1	1.6	55	21.48438	
0	0.041985	#NULL!	29	0	21	0	1.64	60	22.30815	
0	0.053813	#NULL!	30	0	25	0	1.73	76	25.39343	
0	0.063712	#NULL!	28	0	31	0	1.7	61	21.10727	
0	0.06431	#NULL!	29	0	28	1	1.55	43	17.89802	
0	0.058316	#NULL!	29	0	25	1	1.6	47	18.35938	
0	0.061473	#NULL!	28	0	35	1	1.7	60	20.76125	
0	0.048268	#NULL!	30	3	25	1	1.52	42	18.17867	
0	0.058162	#NULL!	28	0	24	1	1.6	49	19.14063	


Edcucati	SFMPQ	感觉总分	情感总分	VAS	现实疼痛	ODI_perc	PCS_0W	PCS_R_0W	PCS_H_0W	
20	6	4	2	6	1	10	19	11	2	
19	12	10	2	6	3	16	7	3	1	
18	11	7	4	6	0	12	9	3	0	
18	6	2	4	8	2	17.5	6	3	1	
16	14	9	5	6	3	24.44	18	7	6	
16	8	4	4	5.8	0	12	9	6	1	
16	2	1	1	4	1	8	3	2	0	
21	11	6	5	8	3	14	15	4	3	
16	7	4	3	4	0	8	10	6	2	
16	3	3	0	7.5	2	22	10	5	2	
12	12	9	3	6	0	28	25	9	6	
17	13	9	4	5.2	2	24.44	15	5	5	
17	6	4	2	3.4	1	17.5	11	6	3	
18	6	4	2	6	1	4	9	4	4	
16	2	2	0	3.4	1	4	1	0	1	
19	6	4	2	6.5	0	17.78	15	7	2	
19	19	12	7	5.7	1	20	14	4	5	
18	7	4	3	6	1	8.89	5	2	2	
17	4	2	2	6	1	11.11	9	7	0	
18	8	5	3	4.8	1	32	19	5	5	
11	9	9	0	6	1	22	3	1	0	
18	11	4	7	6	2	17.78	24	9	6	
18	6	3	3	5.1	1	8.89	3	1	1	
16	4	2	2	6.4	1	12	4	2	2	
15	22	15	7	6.2	1	16	22	5	5	
18	10	9	1	8	2	10	9	5	2	
18	5	3	2	5	1	6	8	6	2	
18	5	2	3	5.6	0	7.78	33	13	6	
17	9	5	4	6	1	12	22	7	5	
18	11	6	5	7.6	2	16	26	21	5	
20	16	10	6	7.5	2	20	24	11	4	
18	22	15	7	6	2	18	20	13	5	
18	6	3	3	7.2	1	12	26	12	6	
18	24	16	8	6	2	18	25	12	5	
17	3	2	1	5.9	3	10	2	0	0	
18	8	5	3	6.8	1	12	2	0	1	
16	13	7	6	7.7	3	33.33	24	7	7	
18	10	7	3	6.1	1	22	15	6	3	
17	8	5	3	4.6	1	22.22	29	11	6	
16	15	10	5	5.7	3	22	31	10	6	
20	5	4	1	5	1	12	6	3	1	
18	6	4	2	6.2	1	10	12	5	3	
16	12	5	7	7	1	10	11	3	3	
23	21	13	8	6	1	14	21	7	6	
18	5	2	3	6.1	1	8	1	1	0	
21	12	6	6	5.5	2	10	17	4	4	
16	9	3	6	4	1	13.33	19	6	6	
22	8	4	4	6.1	1	14	2	0	1	
18	9	5	4	4	1	15	16	7	2	
16	8	3	5	4.1	2	16	14	6	5	
22	7	5	2	4.4	3	26	8	5	2	
22	7	3	4	6	4	22	29	7	15	
17	6	2	4	5.1	0	0	8	7	1	


16	#NULL!	#NULL!	#NULL!	#NULL!	#NULL!	#NULL!	#NULL!	#NULL!	#NULL!	
17	#NULL!	#NULL!	#NULL!	#NULL!	#NULL!	#NULL!	#NULL!	#NULL!	#NULL!	
15	#NULL!	#NULL!	#NULL!	#NULL!	#NULL!	#NULL!	#NULL!	#NULL!	#NULL!	
18	#NULL!	#NULL!	#NULL!	#NULL!	#NULL!	#NULL!	#NULL!	#NULL!	#NULL!	
14	#NULL!	#NULL!	#NULL!	#NULL!	#NULL!	#NULL!	#NULL!	#NULL!	#NULL!	
16	#NULL!	#NULL!	#NULL!	#NULL!	#NULL!	#NULL!	#NULL!	#NULL!	#NULL!	
15	#NULL!	#NULL!	#NULL!	#NULL!	#NULL!	#NULL!	#NULL!	#NULL!	#NULL!	
18	#NULL!	#NULL!	#NULL!	#NULL!	#NULL!	#NULL!	#NULL!	#NULL!	#NULL!	
18	#NULL!	#NULL!	#NULL!	#NULL!	#NULL!	#NULL!	#NULL!	#NULL!	#NULL!	
18	#NULL!	#NULL!	#NULL!	#NULL!	#NULL!	#NULL!	#NULL!	#NULL!	#NULL!	
12	#NULL!	#NULL!	#NULL!	#NULL!	#NULL!	#NULL!	#NULL!	#NULL!	#NULL!	
9	#NULL!	#NULL!	#NULL!	#NULL!	#NULL!	#NULL!	#NULL!	#NULL!	#NULL!	
18	#NULL!	#NULL!	#NULL!	#NULL!	#NULL!	#NULL!	#NULL!	#NULL!	#NULL!	
18	#NULL!	#NULL!	#NULL!	#NULL!	#NULL!	#NULL!	#NULL!	#NULL!	#NULL!	
18	#NULL!	#NULL!	#NULL!	#NULL!	#NULL!	#NULL!	#NULL!	#NULL!	#NULL!	
16	#NULL!	#NULL!	#NULL!	#NULL!	#NULL!	#NULL!	#NULL!	#NULL!	#NULL!	
21	#NULL!	#NULL!	#NULL!	#NULL!	#NULL!	#NULL!	#NULL!	#NULL!	#NULL!	
19	#NULL!	#NULL!	#NULL!	#NULL!	#NULL!	#NULL!	#NULL!	#NULL!	#NULL!	
17	#NULL!	#NULL!	#NULL!	#NULL!	#NULL!	#NULL!	#NULL!	#NULL!	#NULL!	
19	#NULL!	#NULL!	#NULL!	#NULL!	#NULL!	#NULL!	#NULL!	#NULL!	#NULL!	
21	#NULL!	#NULL!	#NULL!	#NULL!	#NULL!	#NULL!	#NULL!	#NULL!	#NULL!	
9	#NULL!	#NULL!	#NULL!	#NULL!	#NULL!	#NULL!	#NULL!	#NULL!	#NULL!	
19	#NULL!	#NULL!	#NULL!	#NULL!	#NULL!	#NULL!	#NULL!	#NULL!	#NULL!	
17	#NULL!	#NULL!	#NULL!	#NULL!	#NULL!	#NULL!	#NULL!	#NULL!	#NULL!	
19	#NULL!	#NULL!	#NULL!	#NULL!	#NULL!	#NULL!	#NULL!	#NULL!	#NULL!	
21	#NULL!	#NULL!	#NULL!	#NULL!	#NULL!	#NULL!	#NULL!	#NULL!	#NULL!	
22	#NULL!	#NULL!	#NULL!	#NULL!	#NULL!	#NULL!	#NULL!	#NULL!	#NULL!	
22	#NULL!	#NULL!	#NULL!	#NULL!	#NULL!	#NULL!	#NULL!	#NULL!	#NULL!	
18	#NULL!	#NULL!	#NULL!	#NULL!	#NULL!	#NULL!	#NULL!	#NULL!	#NULL!	
21	#NULL!	#NULL!	#NULL!	#NULL!	#NULL!	#NULL!	#NULL!	#NULL!	#NULL!	
18	#NULL!	#NULL!	#NULL!	#NULL!	#NULL!	#NULL!	#NULL!	#NULL!	#NULL!	
19	#NULL!	#NULL!	#NULL!	#NULL!	#NULL!	#NULL!	#NULL!	#NULL!	#NULL!	
17	#NULL!	#NULL!	#NULL!	#NULL!	#NULL!	#NULL!	#NULL!	#NULL!	#NULL!	
19	#NULL!	#NULL!	#NULL!	#NULL!	#NULL!	#NULL!	#NULL!	#NULL!	#NULL!	
18	#NULL!	#NULL!	#NULL!	#NULL!	#NULL!	#NULL!	#NULL!	#NULL!	#NULL!	
18	#NULL!	#NULL!	#NULL!	#NULL!	#NULL!	#NULL!	#NULL!	#NULL!	#NULL!	
17	#NULL!	#NULL!	#NULL!	#NULL!	#NULL!	#NULL!	#NULL!	#NULL!	#NULL!	
21	#NULL!	#NULL!	#NULL!	#NULL!	#NULL!	#NULL!	#NULL!	#NULL!	#NULL!	
19	#NULL!	#NULL!	#NULL!	#NULL!	#NULL!	#NULL!	#NULL!	#NULL!	#NULL!	
11	#NULL!	#NULL!	#NULL!	#NULL!	#NULL!	#NULL!	#NULL!	#NULL!	#NULL!	
20	#NULL!	#NULL!	#NULL!	#NULL!	#NULL!	#NULL!	#NULL!	#NULL!	#NULL!	
22	#NULL!	#NULL!	#NULL!	#NULL!	#NULL!	#NULL!	#NULL!	#NULL!	#NULL!	
15	#NULL!	#NULL!	#NULL!	#NULL!	#NULL!	#NULL!	#NULL!	#NULL!	#NULL!	
18	#NULL!	#NULL!	#NULL!	#NULL!	#NULL!	#NULL!	#NULL!	#NULL!	#NULL!	
21	#NULL!	#NULL!	#NULL!	#NULL!	#NULL!	#NULL!	#NULL!	#NULL!	#NULL!	
18	#NULL!	#NULL!	#NULL!	#NULL!	#NULL!	#NULL!	#NULL!	#NULL!	#NULL!	
20	#NULL!	#NULL!	#NULL!	#NULL!	#NULL!	#NULL!	#NULL!	#NULL!	#NULL!	
18	#NULL!	#NULL!	#NULL!	#NULL!	#NULL!	#NULL!	#NULL!	#NULL!	#NULL!	
21	#NULL!	#NULL!	#NULL!	#NULL!	#NULL!	#NULL!	#NULL!	#NULL!	#NULL!	
20	#NULL!	#NULL!	#NULL!	#NULL!	#NULL!	#NULL!	#NULL!	#NULL!	#NULL!	
11	#NULL!	#NULL!	#NULL!	#NULL!	#NULL!	#NULL!	#NULL!	#NULL!	#NULL!	
19	#NULL!	#NULL!	#NULL!	#NULL!	#NULL!	#NULL!	#NULL!	#NULL!	#NULL!	
17	#NULL!	#NULL!	#NULL!	#NULL!	#NULL!	#NULL!	#NULL!	#NULL!	#NULL!	
19	#NULL!	#NULL!	#NULL!	#NULL!	#NULL!	#NULL!	#NULL!	#NULL!	#NULL!	


8	#NULL!	#NULL!	#NULL!	#NULL!	#NULL!	#NULL!	#NULL!	#NULL!	#NULL!	
18	#NULL!	#NULL!	#NULL!	#NULL!	#NULL!	#NULL!	#NULL!	#NULL!	#NULL!	
20	#NULL!	#NULL!	#NULL!	#NULL!	#NULL!	#NULL!	#NULL!	#NULL!	#NULL!	
10	#NULL!	#NULL!	#NULL!	#NULL!	#NULL!	#NULL!	#NULL!	#NULL!	#NULL!	
18	#NULL!	#NULL!	#NULL!	#NULL!	#NULL!	#NULL!	#NULL!	#NULL!	#NULL!	
3	#NULL!	#NULL!	#NULL!	#NULL!	#NULL!	#NULL!	#NULL!	#NULL!	#NULL!	
18	#NULL!	#NULL!	#NULL!	#NULL!	#NULL!	#NULL!	#NULL!	#NULL!	#NULL!	
17	#NULL!	#NULL!	#NULL!	#NULL!	#NULL!	#NULL!	#NULL!	#NULL!	#NULL!	
22	#NULL!	#NULL!	#NULL!	#NULL!	#NULL!	#NULL!	#NULL!	#NULL!	#NULL!	
19	#NULL!	#NULL!	#NULL!	#NULL!	#NULL!	#NULL!	#NULL!	#NULL!	#NULL!	
16	#NULL!	#NULL!	#NULL!	#NULL!	#NULL!	#NULL!	#NULL!	#NULL!	#NULL!	
18	#NULL!	#NULL!	#NULL!	#NULL!	#NULL!	#NULL!	#NULL!	#NULL!	#NULL!	
18	#NULL!	#NULL!	#NULL!	#NULL!	#NULL!	#NULL!	#NULL!	#NULL!	#NULL!	


PCS_M_0W	VBM_Ling	VBM_Caud	VBM_Caud	zFC_Ling	zFC_Ling	GCA_Caud	zFC_Ling	GCA_Caudate_R_y2x	
6	0.383493	0.675807	0.59373	0.040562	-0.03387	-0.08533	-0.0397	0.114798	
3	0.403597	0.550737	0.541555	0.290255	0.560916	0.027305	0.564628	-0.40679	
6	0.635608	0.697595	0.615835	0.181568	0.196511	-0.77016	0.193849	-0.41976	
2	0.470406	0.649178	0.592228	0.119835	0.166785	-0.66999	0.154063	-0.71777	
5	0.437222	0.536934	0.461828	0.042447	0.112314	-0.11331	0.105749	-0.13957	
2	0.462315	0.61801	0.583798	-0.00152	-0.13657	-1.22971	-0.14515	-0.79834	
1	0.520193	0.598671	0.534825	0.328339	0.005747	0.107813	-0.00404	0.584916	
8	0.540076	0.653125	0.546406	0.14175	0.149041	-0.20575	0.142208	-0.16863	
2	0.565724	0.590857	0.499304	0.394162	0.069857	0.399835	0.054508	0.459933	
3	0.426964	0.509191	0.474648	0.426	-0.13966	-1.07226	-0.18734	-1.28318	
10	0.501367	0.535052	0.477342	0.141439	0.061141	-0.39973	0.07078	0.350569	
5	0.45823	0.436756	0.382771	-0.00626	-0.02451	0.109473	-0.02909	0.229047	
2	0.516194	0.69212	0.560919	0.380677	0.516448	-0.25383	0.525385	-0.52384	
1	0.548543	0.660227	0.62403	0.017499	-0.08441	-0.14271	-0.09931	-0.1833	
0	0.557831	0.730106	0.618786	0.22661	0.020749	-0.09375	-0.00251	-0.1516	
6	0.429692	0.641503	0.561775	0.247971	0.287879	0.636228	0.283575	1.047769	
5	0.423653	0.651503	0.600621	-0.03906	0.167069	-0.09914	0.172613	-0.22541	
1	0.471227	0.600811	0.522659	0.492841	0.316978	1.410578	0.312381	1.30011	
2	0.488504	0.699363	0.609408	0.159266	0.111605	0.269877	0.115489	0.021061	
9	0.522204	0.812335	0.749664	0.213029	0.20902	-0.67258	0.19905	-0.88839	
2	0.469851	0.563079	0.512439	-0.20287	-0.05807	-0.40794	-0.06259	-0.21543	
9	0.401363	0.520066	0.486313	0.04045	0.136518	-0.16632	0.12828	-0.433	
1	0.515418	0.536912	0.502184	0.079713	0.017202	0.077956	0.002257	0.144935	
0	0.452702	0.684705	0.56656	0.208339	0.207594	0.24898	0.207884	0.748864	
12	0.549908	0.687165	0.611348	0.191964	0.207122	0.462371	0.215761	0.543823	
2	0.510466	0.60948	0.533297	-0.05919	0.059156	0.048711	0.061085	0.140922	
0	0.388736	0.659848	0.581583	0.42894	0.489844	-0.25038	0.477462	-0.28272	
14	0.5244	0.590164	0.605947	-0.15577	0.010918	-0.00697	-0.00712	-0.02419	
10	0.35482	0.604316	0.583513	0.090131	0.237054	-0.1964	0.23668	-0.24027	
0	0.547215	0.589823	0.586095	0.391619	0.310101	-0.43098	0.298844	-0.2502	
9	0.453647	0.687106	0.678019	0.188477	0.152903	-1.52389	0.175642	-1.61925	
2	0.35304	0.530842	0.442009	0.241504	0.318508	0.40474	0.325776	0.493418	
8	0.495078	0.552843	0.522823	0.021604	0.070406	0.747127	0.063354	0.746123	
8	0.426935	0.547672	0.537347	0.094599	0.248226	-0.2572	0.258525	-0.30787	
2	0.440977	0.636433	0.57955	0.11505	-0.11957	0.365032	-0.11963	0.141372	
1	0.432424	0.536467	0.487335	0.144766	0.266048	-0.62484	0.276399	-0.52706	
10	0.492944	0.723371	0.606982	0.27448	0.117282	-0.69293	0.122446	-1.18907	
6	0.525617	0.657144	0.597782	0.112655	-0.09011	-0.05263	-0.09563	-0.11966	
12	0.475632	0.587228	0.509176	-0.03938	0.08422	-0.87408	0.082041	-0.6291	
15	0.530967	0.676257	0.65322	-0.03112	0.209956	-1.86522	0.200387	-1.70074	
2	0.589633	0.55934	0.527859	0.097065	0.042318	0.174003	0.037746	0.33943	
4	0.526816	0.683596	0.61973	0.555524	0.413793	0.795679	0.407703	1.00936	
5	0.526299	0.717209	0.63816	-0.02148	-0.00195	-1.2337	-0.00811	-1.07973	
8	0.356839	0.419217	0.368011	0.523277	0.397984	0.068872	0.383065	-0.1032	
0	0.505777	0.66017	0.586778	0.03314	-0.00505	0.71422	-0.01241	0.55892	
9	0.526669	0.511777	0.471846	0.287911	0.221754	-1.39027	0.221806	-1.37296	
7	0.452758	0.526956	0.469758	-0.03113	0.012819	-1.22341	0.007784	-1.08133	
1	0.451434	0.527697	0.523353	0.350339	0.291395	1.09557	0.289019	1.513994	
7	0.421322	0.639024	0.572808	0.295685	0.177326	-0.77329	0.184825	-0.91772	
3	0.601803	0.832923	0.700544	0.283171	0.341176	-0.05641	0.339231	-0.07717	
1	0.526109	0.635675	0.617322	0.269709	0.243028	-0.01139	0.23337	-0.15776	
7	0.445572	0.734381	0.554104	-0.2674	-0.22915	0.865787	-0.22888	1.083912	
0	0.499716	0.560859	0.548097	0.018673	0.076813	-0.39838	0.075249	-0.84976	


#NULL!	0.581375	0.73552	0.677716	0.209673	0.288953	0.755974	0.29221	0.881169	
#NULL!	0.497373	0.593336	0.543603	0.367483	0.395813	0.290195	0.396367	0.53089	
#NULL!	0.69075	0.644552	0.571625	0.209305	0.288741	0.75306	0.292047	0.877704	
#NULL!	0.598891	0.698631	0.67253	-0.02497	-0.11834	0.113444	-0.11342	0.14875	
#NULL!	0.533801	0.654541	0.610591	0.259145	0.115873	1.414009	0.111055	1.415265	
#NULL!	0.469817	0.606671	0.505587	0.129322	0.220451	-0.03131	0.209832	-0.05517	
#NULL!	0.50036	0.629423	0.596165	0.152934	0.093454	0.05308	0.079674	0.279662	
#NULL!	0.501635	0.611677	0.558532	0.264782	0.234893	-0.62275	0.238314	-0.39399	
#NULL!	0.586305	0.706842	0.652105	0.317277	0.228225	0.138926	0.222942	0.186772	
#NULL!	0.505586	0.685264	0.61941	0.01517	0.065921	0.532674	0.063994	0.484663	
#NULL!	0.508276	0.457855	0.526449	0.257393	0.135229	0.905717	0.144943	0.963621	
#NULL!	0.525231	0.469134	0.489734	0.25671	0.203498	0.796153	0.205145	1.120427	
#NULL!	0.452594	0.706787	0.627602	0.429786	0.49171	0.713053	0.49593	0.941301	
#NULL!	0.583292	0.65352	0.611266	0.264769	0.20611	-0.58392	0.209396	-0.08456	
#NULL!	0.583457	0.715784	0.622203	0.495886	0.466391	-0.18341	0.470776	-0.08336	
#NULL!	0.608858	0.755943	0.673804	0.311869	0.23974	-0.41686	0.245873	-0.3037	
#NULL!	0.543884	0.744082	0.626088	0.354784	0.370981	1.415959	0.373225	1.672498	
#NULL!	0.573263	0.669134	0.58057	0.346216	0.379437	-0.70935	0.387585	-0.30083	
#NULL!	0.581694	0.815303	0.682021	0.343221	0.251874	0.670178	0.243498	0.590149	
#NULL!	0.534193	0.624967	0.588181	0.021951	0.090132	0.751267	0.084904	0.70526	
#NULL!	0.589352	0.572083	0.531434	0.48515	0.584126	0.710608	0.600194	0.636608	
#NULL!	0.615802	0.681969	0.617979	0.061389	0.068796	1.361539	0.056737	1.274269	
#NULL!	0.520918	0.658633	0.584007	0.351644	0.223528	-0.66794	0.209974	-0.58376	
#NULL!	0.493975	0.705103	0.628661	0.273099	0.310139	0.680966	0.322844	0.528146	
#NULL!	0.522091	0.652909	0.542986	0.380459	0.468006	0.579968	0.4748	0.632436	
#NULL!	0.564588	0.654546	0.598623	0.306519	0.301158	-1.28215	0.292602	-1.64791	
#NULL!	0.527526	0.560979	0.494023	0.137373	0.112172	1.256839	0.116662	1.463569	
#NULL!	0.589433	0.685104	0.624468	0.510898	0.379184	0.571511	0.350807	0.297711	
#NULL!	0.443003	0.579042	0.538251	0.699069	0.670253	-0.261	0.67735	-0.66731	
#NULL!	0.523672	0.799869	0.684604	0.620589	0.596935	0.09591	0.598568	0.151681	
#NULL!	0.555424	0.689549	0.612102	0.531252	0.318611	0.838821	0.305275	0.825094	
#NULL!	0.559058	0.646594	0.593522	0.427849	0.374024	1.50071	0.386722	1.195886	
#NULL!	0.428411	0.715608	0.677878	0.194962	0.238127	1.069042	0.227583	1.205661	
#NULL!	0.451447	0.736442	0.724515	0.638887	0.497838	1.166802	0.517991	1.20728	
#NULL!	0.587252	0.739851	0.669331	0.634358	0.520847	0.688306	0.529166	1.105735	
#NULL!	0.501494	0.597318	0.521537	0.366836	0.378206	1.045472	0.386785	1.081394	
#NULL!	0.514914	0.752389	0.602324	0.10573	0.06213	0.899446	0.067979	1.451604	
#NULL!	0.55738	0.832606	0.654788	0.056922	0.197447	-0.5199	0.186219	-0.55123	
#NULL!	0.614932	0.788589	0.695598	0.16591	0.128314	1.165836	0.128635	1.587256	
#NULL!	0.491112	0.586443	0.540838	0.428015	0.429706	-0.08277	0.434288	-0.31869	
#NULL!	0.515106	0.720239	0.664753	0.02543	-0.02395	0.335475	-0.03707	0.627424	
#NULL!	0.41475	0.642589	0.524509	0.682874	0.556479	1.036117	0.584851	1.209847	
#NULL!	0.523846	0.558862	0.497917	0.302718	0.240006	-0.27879	0.233237	-0.17545	
#NULL!	0.500248	0.819026	0.734605	0.137977	0.107333	0.496758	0.09715	0.760184	
#NULL!	0.56249	0.740227	0.648084	-0.00576	0.110517	-0.38249	0.104452	-0.711	
#NULL!	0.52193	0.655666	0.567001	0.405893	0.425722	1.088896	0.438461	1.033275	
#NULL!	0.460762	0.836052	0.698395	0.18325	0.197352	-0.74128	0.182328	-0.80052	
#NULL!	0.522229	0.617406	0.626807	0.015821	-0.03801	1.077622	-0.04866	1.111317	
#NULL!	0.52043	0.696598	0.595979	0.384491	0.253835	1.182475	0.242409	1.106406	
#NULL!	0.527593	0.692265	0.617131	0.130894	0.154089	0.26387	0.160646	-0.01617	
#NULL!	0.514507	0.714259	0.631935	0.730682	0.663865	0.949056	0.65027	0.934852	
#NULL!	0.546344	0.630423	0.535544	0.251417	0.19116	0.442736	0.189256	0.693469	
#NULL!	0.515015	0.711395	0.584029	0.275883	0.274874	1.628475	0.271688	1.539135	
#NULL!	0.476874	0.761292	0.604263	0.338918	0.210636	-0.12979	0.181368	-0.30829	


#NULL!	0.471835	0.758722	0.669335	0.672798	0.565149	1.066748	0.568062	1.092391	
#NULL!	0.524051	0.573877	0.567495	0.091979	0.241948	0.417053	0.251033	0.31916	
#NULL!	0.380469	0.653337	0.562636	0.05349	0.183956	-0.2942	0.191766	-0.65637	
#NULL!	0.441482	0.610964	0.551574	0.330463	0.351926	-0.26143	0.362467	-0.39102	
#NULL!	0.498441	0.775899	0.635022	0.427726	0.429882	0.076976	0.421996	-0.28621	
#NULL!	0.486238	0.578941	0.599143	0.050161	-0.03563	0.053317	-0.0308	-0.00724	
#NULL!	0.509229	0.63144	0.587901	0.263312	0.086108	1.282468	0.076508	1.277598	
#NULL!	0.555201	0.864794	0.728258	0.791141	0.745626	-0.12294	0.748492	-0.27458	
#NULL!	0.496514	0.752104	0.638169	0.098408	0.097042	0.873321	0.09055	0.867284	
#NULL!	0.505362	0.613457	0.55238	0.356793	0.39396	0.587929	0.398705	0.365798	
#NULL!	0.59766	0.64391	0.561102	0.132841	0.261927	0.09438	0.278933	0.437137	
#NULL!	0.549737	0.710205	0.610483	0.381072	0.398802	-0.40428	0.420809	-0.55571	
#NULL!	0.519223	0.729607	0.673252	0.417954	0.356797	0.915094	0.345833	1.11603	

_CLBP_convariating_headmotion
